# The Question of Data Integrity in Article-Level Metrics

**DOI:** 10.1371/journal.pbio.1002161

**Published:** 2015-08-21

**Authors:** Gregg Gordon, Jennifer Lin, Richard Cave, Ralph Dandrea

**Affiliations:** 1 Social Science Research Network, Rochester, New York, United States of America; 2 Public Library of Science, San Francisco, California, United States of America; 3 Independent, San Francisco, California, United States of America

## Abstract

Interest in and use of article-level metrics (ALMs) has grown rapidly amongst the research community, by researchers, publishers, funders, and research institutions. As this happens, it is critical to ensure secure and reliable data that is trustworthy and can be used by all. Two case studies are presented, which illustrate different approaches to establishing ALM data integrity.

## Abstract 2

Article-level metric (ALM) data need to be secure and reliable if they are to be trusted and used by all. This Perspective explores the different approaches taken by two organizations to establish ALM data integrity.

## I. The Price of Purpose

Scholarly journals had published a cumulative total of 50 million scholarly articles by 2010 [1]. An estimated 114 million scholarly documents currently reside on the web [2]. Moreover, what is considered scholarly research has expanded—boundaries between working manuscripts and accepted papers blur, and scholarly communications take on new forms. Our existing mechanisms for sizing up a research article are sorely inadequate in the face of this acceleration of growth.

The journal in which an article is published has served as the long-standing, default solution, and the Journal Impact Factor (JIF) has become the exclusive proxy for importance in the past few decades. Today, its shortcomings are widely documented [1,3] and its vulnerability to manipulation also well understood [4].

To redress the inadequacies of journal-level measures of article relevancy, article-level metrics (ALMs) were introduced in the 1990s and expanded greatly in the late 2000s to capture signals from the broad spectrum of ways in which researchers engage with articles: disseminating, accessing, commenting, and citing research. This multidimensional suite of indicators includes citations, article usage (online page views and downloads), and altmetrics (social metrics that incorporate bookmarking and dissemination activity, media and blog coverage, discussion activity, and ratings). With its broad suite of indicators, the ALM framework offers more ways to measure research based on individual and community need. ALMs can help researchers find the literature relevant to their interests with a custom selection of relevancy signals. They may also provide more diverse evidence of research engagement for reporting scholarly contributions as funder and institutional interest continues to grow in this area [5].

But with great promise also comes considerable concern as ALMs continue to develop into formal credentials that are widely adopted and incorporated into systems of evaluation. The science of ALMs—the conceptual and technical foundations—is still being established, but there is general agreement on their potential value amongst funders, institutions, researchers, and bibliometricians who continue to test the hypotheses and assumptions that undergird this enterprise [6]. Gaming citations, the intentional or unintentional attempt to mislead the measure, has long been a worry [7,8]. With the introduction of new metrics, the concern has understandably broadened in step with the expansion of measures available.

## II. The Question of Data Integrity

Public Library of Science (PLOS) (http://articlemetrics.github.io/plos/) and the Social Science Research Network (SSRN) (http://www.ssrn.com/en/index.cfm/ssrn-faq/#download) are early ALM providers for their publications. Since 2009, they have been joined by most major publishers. ALM services (ex: Altmetric, ImpactStory, Plum Analytics, etc.) have significantly contributed to the proliferation of interest in these metrics. The growth of the community-owned, open source software, Lagotto, has also fueled greater publisher adoption and display of ALMs (http://lagotto.github.io/status/). On the downstream end, researchers, institutions, and funders have begun to explore ways in which they can make sense and use of the data in their activities.

But the research community largely still lacks sufficient support for tracking research activity at the level of the article. The secure and reliable provisioning of data remains a prominent need. Infrastructure required to support an ALM-enabled future is just beginning to emerge across the scholarly landscape [9]. The ALM community recognizes the need for complete, consistent, available, authoritative, and trustworthy data as ALMs mature and garner value [10–13]. To develop ALMs into a widespread resource, we must sufficiently address the problems of standardized data generation across providers, widespread community provisioning, and data integrity to ensure valid and reliable measures. The attainment of this future state relies on a number of elements: robust technology that scales to the growing volume of articles tracked, information security controls, high availability, and mechanisms that ensure the accuracy and consistency of data over its entire lifecycle.

Data integrity—valid and reliable data—is an issue inherent in all information systems. As long as data are collected, stored, and shared, any metric reliant on these data is vulnerable to internal and external quality issues. Data integrity is especially critical during this stage of ALM development when establishing trust is of paramount importance, even as the methods necessary to ensure this are developing. Invalid, unreliable ALM data from any service may introduce confounding variables and uncertainty during the analysis of this new measurement science.

## III. Strategies

We believe that advancing the importance of data integrity also needs to entail a conversation on practical approaches to addressing this issue. To date, ALM data providers do not publicize their mechanisms to ensure data integrity. PLOS and SSRN will share the strategies currently in place in our respective systems.

Data integrity begins with continual tracking and analysis of data activity across a diversity of channels. Researchers share, comment, and discuss articles through any number of available channels to reach their communities. The proverbial water cooler takes numerous forms on the web, spanning platforms dedicated to scholarly activity (Mendeley, F1000Prime, CrossRef, etc.) and mainstream, public sites (Wikipedia, Twitter, etc.). All these are part of the ALM ecosystem.

For both SSRN and PLOS, the strategy centers on usage statistics. Activity largely originates at the source where the article is accessed before it is shared, discussed, and cited. The record of page views and downloads loosely serves as an abstraction for baseline activity, which can be checked relative to the rest of the ALM suite. SSRN focuses on preventative antifraud measures as part of content access regulation, while PLOS focuses on activity detection as part of content delivery.

### The SSRN Experience

In 2000, SSRN began collecting data about each PDF download request and analyzing the activity patterns. It quickly became apparent there was a significant amount of questionable activity through pattern recognition, which was also influencing its “Top 10” rankings list. In response, and in the absence of any industry-standard rules, SSRN established a set of “fraud rules,” which were applied at the time that each download was requested. Initial rules determined if a paper was downloaded more than once from the same IP address within two hours and ensured that the download wasn’t coming from a search engine. If the request passed the fraud rules, the download would be counted. If the request failed the fraud rules, the paper was still delivered to the user making the request, but the download was not counted. These first fraud rules removed most of the inadvertent duplicate counting of downloads due to user error or to reasonable user behaviors that resulted in downloading the same file multiple times.

**Fig 1:**
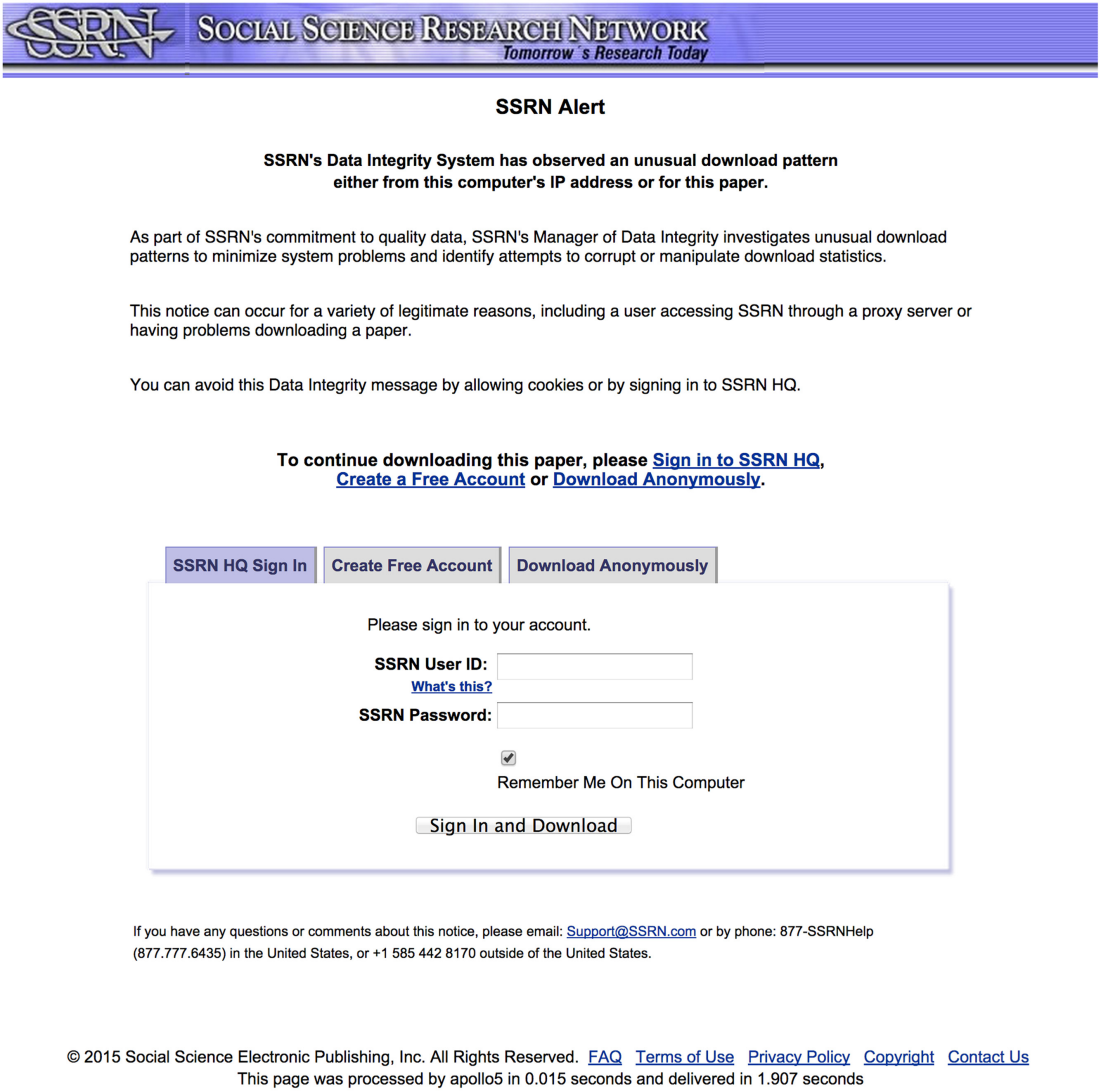
SSRN fraudulent alert.

In 2007, SSRN introduced new “fraud gate” measures based on the user’s IP address and other data. For example, if the same paper was downloaded a number of times from the same IP address, or the number of papers downloaded from a given IP address exceeded a certain limit, then that IP address was placed on a watch list. In 2010, these rules were expanded based on additional data and “behavioral blacklisting” email spam research [14]. Any future download requests violating the rules would result in a message indicating that “potentially fraudulent activity has been detected.” The user would then have three options: sign in using their existing SSRN account, create a new account at SSRN, or accept that the download would not be counted (Fig 1).

**Fig 2:**
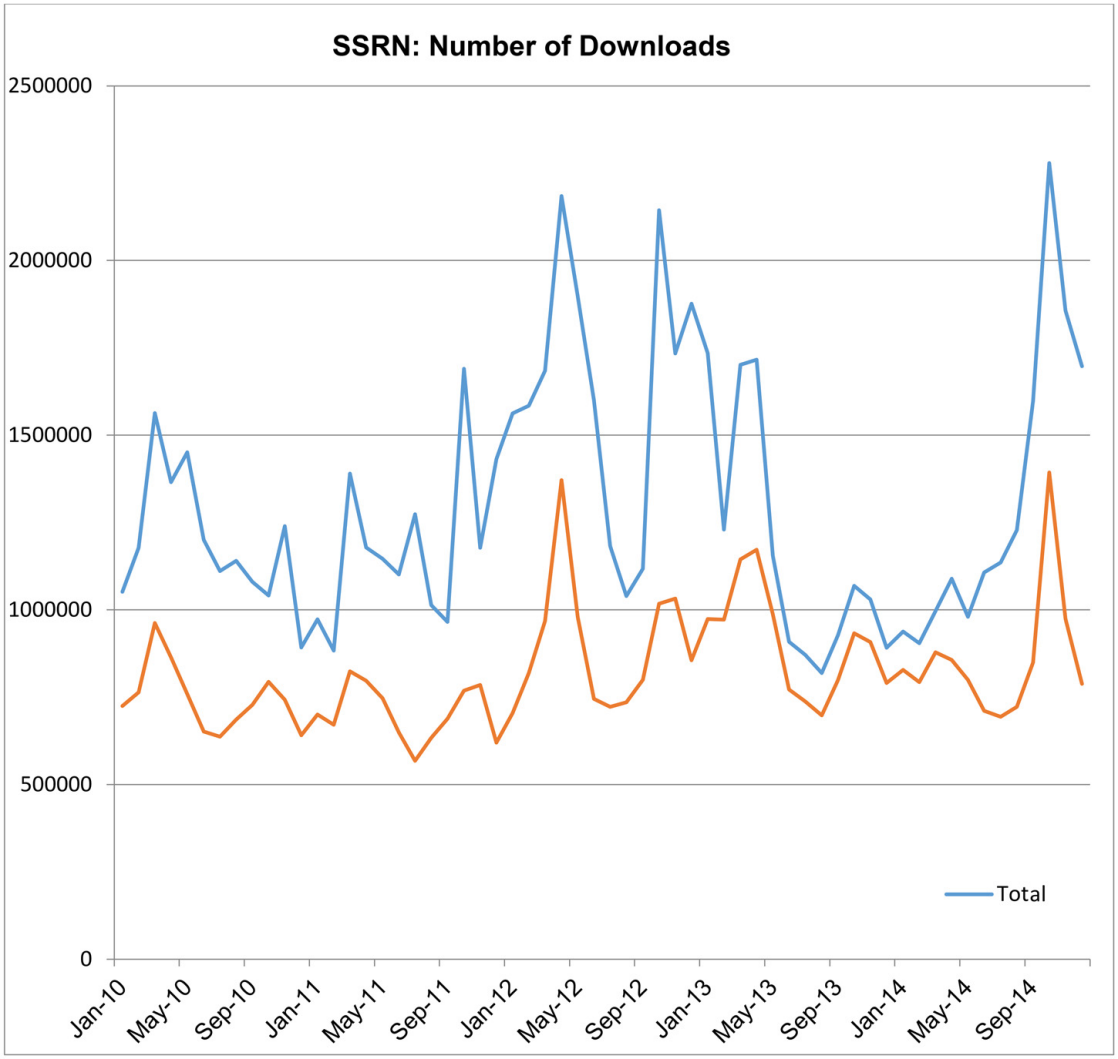
SSRN fraudulent activity detected.

The IP-based fraud gates worked so well that most instances of what was, in retrospect, potentially fraudulent activity ceased immediately. Users that had been identified with the inflation of download counts stopped once it became clear that their activity was being monitored. Since the introduction of the fraud rules, the number of downloads counted by SSRN is much less than the actual number of downloads served. Today, even “questionable” downloads are excluded from a paper’s metrics. For some months, the downloads that have been excluded can exceed the number of downloads counted by SSRN (Fig 2).

SSRN cannot definitively ascertain that the difference between the total downloads and what is counted is due to intentional fraudulence per se. However, particular patterns may suggest this as a potential occurrence:

- a registered user is downloading a paper that they have already downloaded
- the same IP address is downloading a paper multiple times in a given time period
- the IP address is associated with a known search engine
- the IP address is flagged as having potentially fraudulent activity in the past.

These checks provide additional security measures to ensure that SSRN usage counts are valid.

### The PLOS Experience

Since 2009, PLOS has tracked usage for its articles through online views, PDF downloads, and XML downloads on its site. These usage statistics adhere to COUNTER (http://www.projectcounter.org), the scholarly publishing industry standard for recording and reporting online usage statistics. Other article activities tracked include scholarly citations across various indices and a vast range of social web activity (http://alm.plos.org/sources).

The overall approach begins with the proper and complete transfer of the data on article events from the web services where the activity occurred. The PLOS implementation of the Lagotto ALM software provides two levels of checks: a) a dedicated error tracking service to monitor overall system performance and b) filters, an additional layer of scripts that monitor the data collected to detect unusual activity. At the first level, PLOS conducts system error tracking, which requires more detailed monitoring of exception events. Network errors are common, due to the varying states of the many web services involved (e.g., overloaded source server, rate limits exceeded, query time outs, etc.). The system sends an email every 24 hours that summarizes all errors for human follow-up. At the second level, PLOS runs a set of scripts for tracking outliers and pattern recognition analysis to detect unusual activity (usage surges, decreases in cumulative counts, etc.). By tracking typical monthly article activity for a group of articles, a threshold is determined to detect outliers. An algorithmic script is then used to find and identify the outliers. This strategy makes reporting straightforward (Table 1) and provides an efficient approach to monitoring at scale.

**Table 1:** PLOS page view increases. Dec 10, 2013 to Jan 10, 2014.

**10.1371/journal.pone.0080347**	**First Partial Skeleton of a 1.34-Million-Year-Old Paranthropus boisei from Bed II, Olduvai Gorge, Tanzania**	**34,315**
**10.1371/journal.pmed.0020124**	**Why Most Published Research Findings Are False**	**14,252**
**10.1371/journal.pone.0069841**	**Facebook Use Predicts Declines in Subjective Well-Being in Young Adults**	**13,398**
**10.1371/journal.pone.0010894**	**Exon Exchange Approach to Repair Duchenne Dystrophin Transcripts**	**11,453**

**Fig 3:**
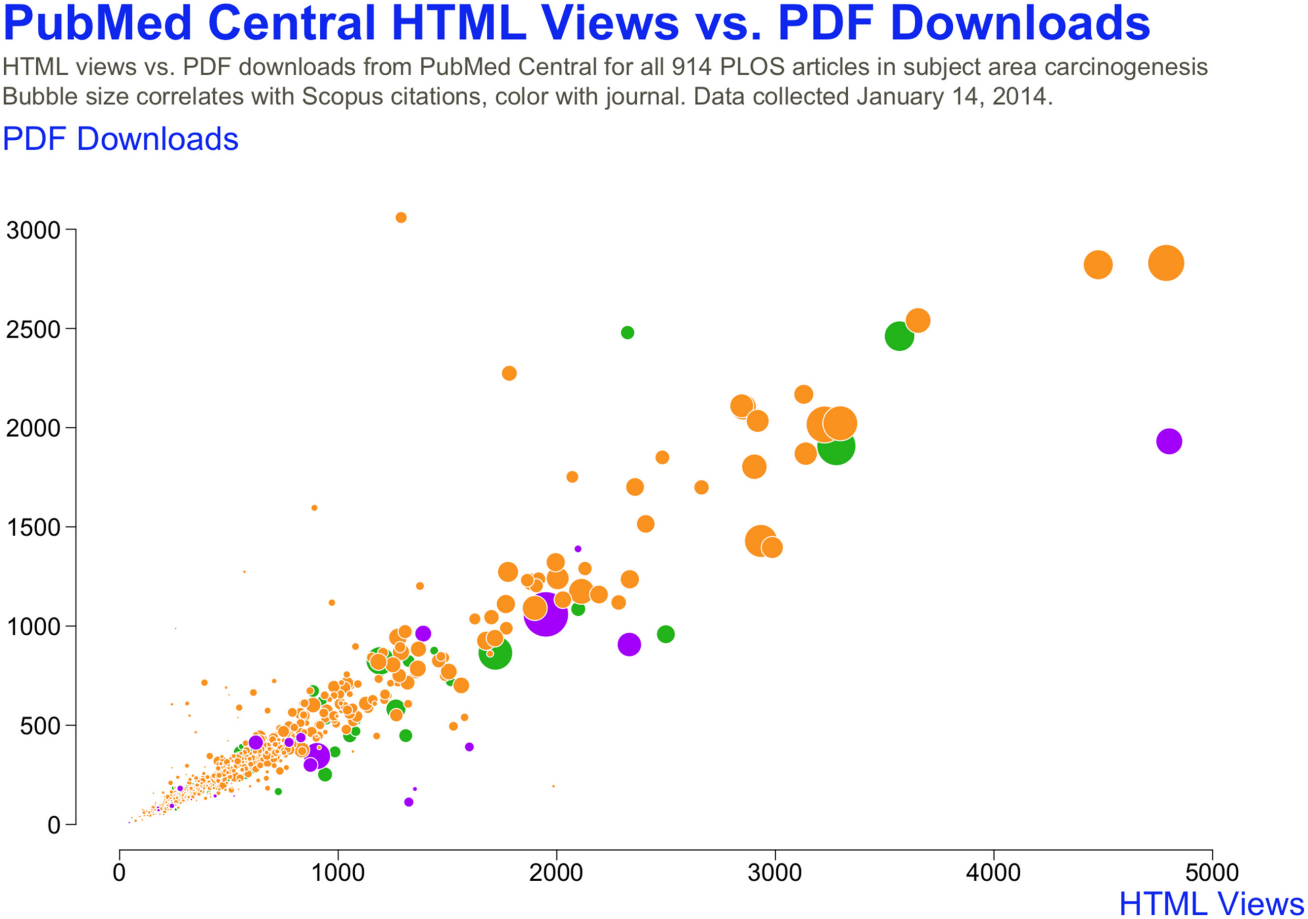
PLOS comparison of PMC page views and downloads.

Pattern recognition can be used to detect gaming by reviewing usage activity across multiple sources and provides a more consistent basis for detection. Article views and PDF downloads on PLOS journals along with usage activity on the PubMed Central article repository reveal general patterns of usage over time (Fig 3). In general, page views of a research article will track closely to the number of PDF downloads. When usage statistics fall outside of the correlation, further verification of higher usage statistics is then needed.

**Fig 4:**
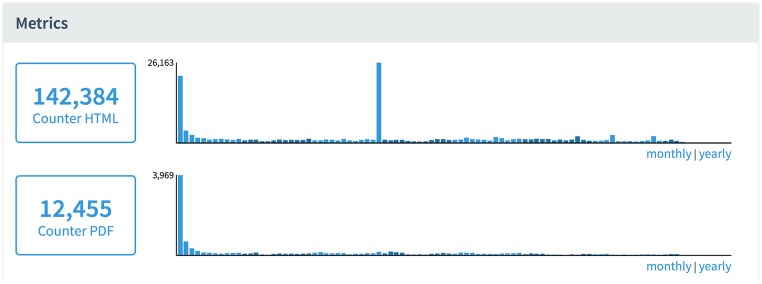
*PLOS Medicine* article with irregular page view activity.

PLOS also cross validates usage activity with other sources. While a direct correlation between the two activities is not given, an article with high usage activity generally exhibits related activity in another metric. Articles with an abnormal spike in one usage activity without a corresponding spike in another are examined to determine the cause of the discrepancy. Fig 4 shows an article with a typical distribution pattern of usage activity until a significant spike occurred three years after publication. Further investigation may then determine the source of the increased usage. In this instance, article referral traffic reveals that the spike in usage activity was due to a blog post on Nature Blogs that discussed the article, rather than fraudulent activity.

But irregular usage only sometimes correlates with higher traffic driven by other sources. PLOS employs a number of tactics to address and prevent systematic abuse, including more detailed analyses of specific users or IP addresses. Repeated hits from a single IP address (human or machine) in server logs are considered artificial inflation of counts. To ensure valid counting of usage data in this case, PLOS adjusts direct activity accordingly for the article in question from the offending agent.

As a publisher where usage activity is occurring on its own platform, PLOS has a wider scope of means available to ensure data integrity, adhering to proper guidelines of collection and exclusion of counts, as well as correcting counts as appropriate when irregularities are found. But data stewardship entails different degrees of responsibility for each agent according to its role. For the data aggregator who is pulling counts of activity from external sources, data stewardship entails ensuring that ALM data are correctly aggregated. Oversight here is limited to ensuring full and accurate integration of data and complete provisioning of fresh data. PLOS serves both roles for the articles in its corpus—the original source for usage data and the aggregator for all external sources.

The efforts summarized above aim to mitigate some of the concerns about data integrity, but more work needs to be done. Currently, usage profile is static and constructed based on historical patterns. Monitoring thresholds built into the Lagotto filters are also static and not ideal in the face of evolving online behaviors. But there is great potential for progress in this area. The application of statistical computing and visualizations for data integrity is currently being tested by data scientists and shows significant promise [15]. Open source software, which extends the ALM technology toolkit in this area, would significantly contribute to the programmatic operationalization of data integrity across data providers.

## IV. Call to Action

ALMs are spreading as the data become more interesting to the scholarly community. They have been found to be valuable for researchers searching for relevant content, for authors interested in highlighting the reach of their work, and for institutions and funders monitoring the progress of the research they support. For any data to be a valuable part of the research information ecosystem, confidence in the metrics is fundamental. ALMs are no different.

The distributed ALM information environment is a network made up of multiple agents that capture activity originating on their site, data aggregators who collect the data, and distributors who enrich and package the data along with any host of additional intermediaries. To understand data integrity from a network standpoint, we recognize the diversity of players and complex information exchanges across the web that occur at each and every site. In the networked world of the scientific enterprise, data integrity is a shared responsibility of all the players involved.

Policies, processes, and supporting technologies are an integral part of a holistic approach and require the endorsement and participation of the entire scholarly community. ALM providers need to clearly describe how they calculate their measures as well as provide an understanding of how they have kept their data free of error, both intentional and unintentional. Shared conventions for data irregularity resolution (excluded sources, data adjustment practices) are necessary for data quality across providers. Individual users can then determine if a particular ALM is trustworthy and reliable enough to inform their work. Funders and research institutions can play a role in demanding that the data be made available in a standardized fashion across publishers and in a replicable and verifiable manner so that the community can scrutinize its strength and trustworthiness. The entire community can establish guidelines and incentives for good and fair behaviors impacting the collection and use of metrics.

So as to be readily monitored, ALM data need to be open, transparent, and auditable. Academia currently operates well with its existing ethos of community scrutiny for plagiarism or data manipulation. Similar community norms are needed for ALM gaming where willful manipulation ought to be treated as a professional offense and reported to respective institutions for further action. In addition to transparency, accountability is a key ingredient to trust. And as chances of discovering gaming increase, the risk of engaging in such practices would be magnified, thereby lowering the incentive. But the question is not just about gaming. The entire research community has an interest in ensuring the highest level of ALM data integrity at large. This path begins with the first step: taking a broader view of what is required for trust and engaging the entire community to consider their role within this ecosystem.

## Abbreviations

ALM: article-level metrics
JIF: Journal Impact Factor
SSRN: Social Science Research Network
